# Surgical and Perioperative Considerations for the Treatment of Cataract in Eyes with Glaucoma: A Literature Review

**DOI:** 10.1155/2021/5575445

**Published:** 2021-04-26

**Authors:** Kleonikos Tsakiris, George Kontadakis, Panagiotis Georgoudis, Zisis Gatzioufas, Athanasios Vergados

**Affiliations:** ^1^Whipps Cross University Hospital, Barts Health NHS Trust, Whipps Cross Road, London E11 1NR, UK; ^2^Department of Ophthalmology, Basel University Hospital, Basel 4051, Switzerland

## Abstract

Cataract surgery in the presence of glaucoma poses certain challenges that need to be addressed to offer the maximum benefit without complications. In this paper, we are reviewing the preoperative assessment, surgical options, the planning, and postoperative care. Cataract surgery can help reduce the intraocular pressure alone or combined with MIGS. When performed in patients with glaucoma, it can transiently increase the intraocular pressure and later on decrease the IOP to levels lower than the postoperative. The preoperative IOP and biometric characteristics are the main predictors of the postoperative course of IOP. The combination of cataract surgery with trabeculectomy remains controversial, in terms of best timing of each operation.

## 1. Introduction

Glaucoma has been ranked as one of the most common causes of visual impairment in the adult population worldwide. In people aged 50 years and older, the leading causes of blindness were cataract followed by uncorrected refractive error and glaucoma in 2015, with increasing prevalence since 1990. It has been calculated that 3.54% of the global population suffers from glaucoma. The vast majority of those cases account for open angle glaucoma (3.5%) and the rest accounts for primary angle closure glaucoma (0.5%). The number of people with glaucoma worldwide aged 40 to 80 years is expected to increase from 64.3 million in 2013 to 111.8 million in 2040 [1, 2].

With glaucoma being a widespread eye disease in the aged population, treatment of cataract needs to be planned while facing glaucoma as a comorbidity and vice versa. Our steps in the management of both eye conditions when coincident often need to be modified to achieve the best outcome for the patient. The cataract operation in such patients is done with special measures depending on the type and stage of glaucoma. Additionally, the IOP lowering medications prescribed to the patient often need to be adapted postoperatively. Combined techniques that manage both conditions simultaneously are also available in the surgical armamentarium and techniques have been developed, but the selection of the most suitable for each case demands careful consideration and is often controversial. It is known that the cataract operation has an IOP lowering effect and that in specific cases of glaucoma might be even the desired one to achieve a therapeutic effect [3].

## 2. Cataract Operation and Intraocular Pressure

Numerous studies have assessed the effect of cataract extraction on the IOP of the operated patients. The effect of the operation in IOP can be divided into 3 categories, the intraoperative effect, the short term postoperative effect, and the long-term effect. Each one needs to be taken into consideration in glaucoma patients.

During phaco surgery, it can be calculated that for every 15 cm of bottle height above the patient's eye level, there is a raise of 11 mm Hg in intraocular pressure (IOP) [4], when there is irrigation without aspiration. Consequently, when the bottle height is adjusted at 1 meter, the IOP can reach as high as 70 mmHg which is around the closing pressure of the central retinal artery. Particularly in eyes with end-stage glaucoma, this could lead to severe vision loss, a phenomenon termed as wipe-out syndrome, that has been described, albeit rarely, after phacoemulsification surgery [5]. In eyes with end-stage glaucoma, it is safer to decrease bottle height and avoid keeping the phaco tip in the anterior chamber without active aspiration during surgery, so that IOP does not reach very high levels.

One of the most frequent complications after phacoemulsification surgery is the spikes of IOP in the immediate postoperative period [6]. The IOP after surgery can raise for several reasons, with the most common being the retained viscoelastic. Residue viscoelastic may cause obstruction of aqueous outflow and increase of IOP postoperatively as reported as early as 1983 [7]. For a given concentration of the viscoelastic in the AC, the lower the viscosity, the higher and more prolonged the IOP spike. The different viscoelastics demonstrate slightly different behaviours regarding the postoperative effect on IOP depending on their properties, such as molecular charge, chain length, and rigidity [8, 9]. The cohesive viscoelastics have larger molecular chains and are aspirated more easily, but may cause higher IOP spikes than the dispersive if not removed, although differences are not great [10]. On the other hand, the use of dispersive viscoelastic is responsible of this effect more often, since it cannot be easily removed from the eye without meticulous aspiration.

Other sources of IOP spikes after cataract include surgical trauma, prolonged surgery, retained lens debris, iris pigment scattering, inflammation, and hyphema [11]. Posterior capsule rupture, vitreous prolapse, and IOL placement in sulcus are considered risk factors for high IOP [12]. Other significant preexisting risk factors are glaucoma, pseudoexfoliation, tamsulosin intake, and myopia [13–18]. The longer axial length and the shallow AC are associated risk factors [19].

The immediate postoperative spikes usually happen 4 to 12 hours postoperatively. Very few patients developed IOP >30 mmg at 24 hours postoperatively, although hypertension can last up to a week [8, 11, 20]. In a study of eyes without glaucoma, IOP spikes of up to 68 mmHg were observed and 7.8% of eyes developed spikes of more than 40 mmHg 4 to 6 hours postoperatively [21], although most of the eyes that develop IOP spikes after cataract surgery will decrease to normal up to 24 hours postoperatively [20, 22].

Glaucoma is considered a risk factor for the development of an IOP spike after cataract surgery, and this has been demonstrated by studies that show increased incidence of early postoperative increase in IOP in eyes with glaucoma in comparison to normal eyes. IOP spikes over 30 mmHg were found in approximately 13% of eyes 1 day postoperatively in a study of eyes with POAG, and that was higher than the percentage found in nonglaucomatous eyes [17]. In a study that sets the threshold at 28 mmHg, the percentage in glaucomatous eyes was 46.4% versus 18.4% in nonglaucomatous [23]. A retrospective study of 271 eyes has shown that 17% of the operated eyes developed an IOP increase of at least 50% in comparison to the preoperative. Among glaucomatous eyes, those with increased risk are the cases with higher axial length and deeper anterior chamber, those that had required an increased number of antiglaucoma medications preoperatively, and those that had required preoperative laser trabeculoplasty. The protective effect of oral acetazolamide as adjunctive treatment in cataract surgery has been recognised in this study [13].

Prevention of IOP spikes is achieved with topical IOP lowering medication or more frequently with administration of oral acetazolamide [24]. This can be done preoperatively or postoperatively. According to a recent study, oral acetazolamide administration 1 hour preoperatively significantly reduced the IOP elevation from 1 to 24 hours postoperatively, while administration 3 hours postoperatively reduced the IOP elevation at 5 hours or more after surgery [25]. In eyes with glaucoma, the AC paracentesis has been shown to be successful as well, although in otherwise healthy eyes it has been demonstrated as having a nonlasting effect [21]. If left untreated, the IOP spikes in otherwise normal eyes might not be harmful for the visual fields according to clinical data [26], but for already compromised glaucomatous eyes, a significant albeit transient postoperative raise in IOP can be detrimental [27].

Apart from these reasons that lead to IOP raising up to 2 days postoperatively, there is a late postoperative risk of IOP rise due to steroid use after cataract surgery [28, 29]. Mostly myopic eyes but also others can be steroid responders, meaning that their IOP might raise most commonly 10 days to 2 weeks after starting topical steroid use. However, steroid induced IOP rise might appear as early as 5 days before surgery and up to several weeks later [28]. This effect is temporary but might be devastating if not timely detected and controlled in case of patients with glaucoma.

Albeit rising the IOP in the early postoperative period, it has been demonstrated by numerous studies that in the long term phacoemulsification has an IOP lowering effect to a variable extent. This result has been observed in both normal and glaucomatous eyes.

The most prominent lowering effect has been observed in eyes with narrow angle glaucoma. The angle configuration is the initial source of hypertension in such eyes, and the removal of the lens offers sufficient space for the iris to retract and increase the angle width. Progression of lens thickness with age is disproportional to the progression of total eye volume which seizes to increase usually in a young age. Consequently, the lens over time takes over more space in the anterior segment and contributes to IOP rise [30]. Removal of the crystalline lens and placement of a much thinner intraocular lens in all operated eyes lead to deepening of the anterior chamber and increase in angle width, an effect with increased clinical significance in eye with narrow preoperative angles. In addition, cataract surgery results in less IOP fluctuations in such eyes [31]. The amount of the effect is correlated with the preoperative IOP and the depth of the AC in such eyes. Other parameters associated to the effect are the lens thickness and the gonioscopy score [32, 33]. The implementation of cataract surgery as a treatment of choice instead of peripheral iridotomy for primary angle closure glaucoma has been highlighted in the literature [34, 35].

The effect of cataract surgery on the IOP is also detectable in eye with open angles. A proposed mechanism for that is the increased posterior traction of the zonules to the ciliary body and the scleral spur due to the posterior displacement of the anterior capsule postoperatively [36]. According to this theory, this traction expands the trabecular meshwork and improves the aqueous outflow. An IOP raising effect of the aging crystalline lens also contributes to this effect [30]. The lowering effect is more prominent on eyes with an increased preoperative IOP and it depends on the biometric characteristics of the eye.

Several studies have looked into the predictive factors for the IOP lowering effect of cataract surgery. Issa et al. developed an index of the pressure to depth ratio in order to predict the lowering effect according to which the IOP reduction was positively correlated to the preoperative IOP and inversely related to the anterior chamber depth [37]. Liu et al. also suggested a formula that was based on IOP and ACD for eyes with ACG [38]. Most studies agree that if preoperative IOP is more than 20 mmHg, the IOP reduction after cataract surgery would be likely significant. Perez et al. in their formulas include as predictors the preoperative IOP in combination with other parameters such as anterior chamber depth, lens thickness, gonioscopy score, and glaucoma status [39].

## 3. Phacoemulsification Surgery in Eyes with Glaucoma: Surgical Technique Considerations

In eyes with primary open angle glaucoma, no special technique modifications are needed usually. In case of terminal stage glaucoma patients care should be taken to avoid significant increasing of the intraoperative IOP. In all patients with glaucoma, especially those that are in view of a possible surgery, the incisions should be placed in clear cornea to avoid damage to the conjunctiva, in order not to compromise future glaucoma surgery.

In patients with PXF, surgery might be demanding due to the zonular instability that many of these patients have. The weakened zonules might lead to zonular dehiscence intraoperatively and need for special measures in order to avoid complications. Additionally, pupil dilation in patients with PXF is often compromised. In patients with PXF glaucoma aspiration of the exfoliation material from the angle at the end of surgery might be beneficial for the postoperative IOP.

In eyes with angle closure, glaucoma surgery is always challenging due to the anatomical characteristics of those eyes and the effect of glaucoma and previous attacks of acute angle closure. Those eyes have shallow anterior chambers that incommode surgical maneuvers and also are often prone to intraoperative choroidal effusion. Posterior synechiae, poor dilation, weak zonules, and low endothelial cell count might complicate surgery furthermore. Surgery needs to be undertaken with caution. Preoperative administration of mannitol to reduce hyaloid volume might be helpful, as well as preoperative administration of acetazolamide to reduce IOP. Care must be taken in the construction of the wounds in order to avoid intraoperative iris prolapse. Maintaining a stable chamber by viscoelastic infusion prior to removal of irrigation handpiece from the eye during surgery is considered to protect from anterior chamber collapse and choroidal effusion in very short eyes. In eyes with extensive anterior synechiae, cataract surgery would be best combined with goniosynechiolysis, in order to separate the anterior synechiae from the trabecular meshwork and achieve more sufficient IOP control. In general, cataract surgery has been proven to be a sufficient first-line treatment for angle closure glaucoma, more effective than laser iridotomy and could be considered as an alternative to this.

## 4. Surgical Considerations in Patients with past Trabeculectomy

It has been well described that cataract surgery in patients with glaucoma is more complex than the routine phacoemulsification. Moreover it is still widely thought that cataract surgery following trabeculectomy will increase the risk of bleb failure [40] in spite of studies claiming otherwise [40, 41]. In fact some surgeons went as far as using cataract surgery in an attempt to treat postoperative hypotony and its complications with good success [42].

It is true that phakic patients with a functioning trabeculectomy will eventually need cataract surgery, especially considering that filtrating surgery is a risk factor for lens opacification. Eventually 50% of patients that underwent this kind of procedures will present with visually significant cataract over the next five years [43].

Taking into consideration this fact, we need to adjust our strategy to the specifics of trabeculectomised patients. Husein et al. showed that early cataract operation has higher incidence of trabeculectomy failure, considering a period of two years gap between the two operations being the safest option of those studied. Additionally, preoperative high intraocular pressure was deemed as a bad prognostic factor, presumably owing to an already malfunctioning bleb [44]. Some authors in fact have proposed the use of anterior segment optical coherence tomography (AS- OCT) [45] and ultrasound biomicroscopy (UBM) [46] to distinguish between well and not-so-well functioning blebs.

It seems clear that the postoperative inflammation induced by phacoemulsification is considered the main factor leading to bleb failure. Action therefore must be taken to perform the surgery in an atraumatic way, including minimal manipulation and a temporal main port incision all whilst maintaining anterior chamber stability. In the case of a malfunctioning bleb, a combined bleb revision approach could be considered [47]. Aggressive anti-inflammatory treatment intra- and postoperatively is adequate, with intraoperative injection of dexamethasone, 5 fluorouracil along with intensive steroid drops postoperatively being some examples [48].

Another risk that needs to be considered is the complete wipe out. Considerable variation exists between the glaucoma specialists as far as the estimated risk is concerned. It has been reported to be higher than 1/100 to even lower than 1/1000. A current UK-based study by the NHS Health Research Authority is hoping to shed more light on this subject [5].

## 5. Minimally Invasive Glaucoma Surgery and Phacoemulsification

Over the past years we have witnessed the emergence of a number of techniques and devices that try to tackle the main problem of trabeculectomy surgery, which is none other than its safety profile and invasive nature. These techniques aim to either increase aqueous outflow either bypassing (e.g., Xen) or enhancing anatomical structures (e.g., gonioscopy assisted transluminal trabeculotomy (GATT), Hydrus, iStent^©^) or decrease aqueous production by cyclodestructive procedures such as endocyclophotocoagulation and micropulse cyclodiode laser.

MIGS are essentially a category of procedures that offer a higher safety profile but lower efficacy than trabeculectomy. The most usual clinical scenario is mild to moderate glaucoma which is either uncontrolled by drops or aims to reduce drops dependency, usually due to compliance issues pertaining to individual patient factors such as lifestyle, frailty, and drop side effects among others [49].

### 5.1. Phacoemulsification Alone

Phacoemulsification is a recognized modality for treating angle closure glaucoma and it can be even considered without the presence of visually significant cataract in clear lens extraction [50]. It has also been proven to lower the intraocular pressure in open angle patients even when performed as standalone. Its effects, albeit not permanent, should not be overlooked, since it is shown to decrease IOP by 5.1% in three years [51].

### 5.2. iStent and iStent Inject^©^

These two devices represent the two generations of a heparin-coated nonferromagnetic titanium stent which when inserted into the trabecular meshwork drain fluid directly to the canal of Schlemm, and their technical characteristics are beyond the scope of this article. Another advantage of iStent inject^©^ is that it comes preloaded with two stents which further increases its efficacy. When combined with phacoemulsification, it has been proven to be more effective than phacoemulsification alone and it reduces the dependency to eye drops [1, 52–55]. Its major advantage however is the easier technique and excellent safety profile, as it was intended for the general ophthalmologist and not the glaucoma specialists alone.

### 5.3. Hydrus

This device, which is a crescent-shaped scaffold is made of nitinol (a nickel-titanium alloy) that is placed on the Schlemm's canal [56]. In a randomized control trial that compared phacoemulsification alone versus combined phaco/hydrus, it was found that 80% of patients that underwent the combined procedure had lower IOP and 73 % was free of drops in two-year follow-up period. The safety profile of the combined versus the standalone cataract operation was the same, besides 1-2 mm of focal peripheral anterior synechiae with no further implications [57].

### 5.4. Gonioscopy-Assisted Transluminal Trabeculotomy (GATT)

It is not very frequent that a name is so descriptive that leaves so little to imagination. GATT is a development of the ab externo trabeculotomy thus salvaging the conjunctiva and sclera, using a Swan Jacob gonio lens and either an illuminated probe or a suture. When combined with phacoemulsification, it reduced the eye pressure from a mean of 23.9 mmHg to 15.5 mmHg and drop dependency from 2.9 to 1.0 during the first 12 months of follow-up. Hyphaema was the only side effect reported in 20% of patients that had the combined procedure. In patients that had standalone GATT choroidal folds, CMO and IOP spikes were seen [58].

### 5.5. Endocyclophotocoagulation (ECP)

ECP is a recognized technique with a diode laser targeting directly on the ciliary processes, with minimal destruction of surrounding tissues and a greatly improved safety profile. When combined with cataract surgery, it can produce a modest yet significant drop in pressure. It was found to reduce the mean IOP from 18.7 mmHg to 14.0 mmHg in 106 eyes of 99 patients after three years of follow-up but with a failure rate of 60%. While this may look disheartening, the vast majority of patients were managed with drops and SLT and only seven ended up needing filtration surgery [59]. Similarly Francis et al. found a decrease of IOP by 13.6% in three years of follow-up [51]. On the downside, endoscopic surgery is a new skill for ophthalmologists that needs ad initio training, and the postoperative inflammation requires intensive anti-inflammatory drop regime.

A very similar but ab externo technique is micropulse cyclophotocoagulation laser. While no publications exist to date dealing with combined micropulse cyclophotocoagulation/phacoemulsification procedures, it has proven to be a safe and effective, minimally invasive treatment. It works by applying short bursts of energy (0.5 sec) followed by rest periods of 1.1 sec. Reported success rates range from 72.7 to 89.5% [59, 60].

### 5.6. Trabectome Combined with Cataract Surgery

Trabectome is a device known to be effective in lowering intraocular pressure with reported evidence since 2005. When combined with cataract surgery, it is found to cause an increased incidence of postoperative cystic macular oedema in comparison to the cataract alone group. However no effect was found in the postoperative refraction and it did not seem to affect the targets set by the surgeons. As such it is still considered to be a viable option [61].

In contrast to the aforementioned techniques and devices, Xen45 and InFocus are bleb forming procedures. When it comes to Xen, it has shown to be effective in reducing pressure and drop dependency quite significantly. Furthermore it has shown to have reduced effectiveness in non-Caucasian patients and when combined with cataract surgery. On top of that it was found to have a high reoperation rate of 37.7% [62]. It has to be noted here that Xen implants are targeting mild to moderate glaucoma patients and cannot usually lower the pressure below mid-teens [63].

On the other side of the spectrum PRESERVFLO^®^ MicroShunt (previously known as InnFocus MicroShunt) which is made of poly(styrene-block-isobutylene-block-styrene), or SIB—a biocompatible, bioinert material—is a bleb forming device which aims to replace trabeculectomy. It has, in fact, a good safety profile and a reduced operating time [64]. In contrast to what is known for trabeculectomy and Xen implants, in a study from the Dominican Republic, there was no significant difference in the drop of pressure between the patients having MicroShunt alone and those having combined Phacoemulsification with MicroShunt surgery [65].

Another device that is worth mentioning is Cypass. This device presented the novel approach of being inserted in the suprachoroidal space, giving a reduction of intraocular pressure of about 20% [66]. In fact it showed superiority over the iStent when combined with cataract surgery. It gained FDA approval following the 2-year long COMPASS study, but it was recalled from circulation following the reduction of endothelial cell count at the 5-year review of the initial study patient cohort [67].

## 6. Toric and Premium Intraocular Lenses in Patients with Glaucoma

Various studies have shown the advantage of the use of toric lenses in cataract patients. Indeed as IOL technology follows the demand of a sharper vision and spectacle free life, patients with glaucoma are no exception to that. It has been shown that toric lenses improve postoperative refractive outcomes in glaucomatous patients. Controversy still exists in patients with short axial length, however, because of biometry unpredictability and change of capsule, so there is a high risk of axis change [68].

Premium lenses on the other hand are less recommended. As a general rule, they decrease the quality of vision in patients with moderate disease. They have been successfully implanted in patients with very early glaucoma which is thought unlikely to progress and in patients with ocular hypertension or glaucoma suspects without disc damage or visual field loss [31]. In more detail aspheric lenses have shown conflicting evidence when it comes to contrast sensitivity. Blue filtering lenses show no difference in contrast sensitivity. Regarding the multifocal IOLs, they have been found to invariably decrease the contrast sensitivity to a greater extend, and even more for the near than the distance. Finally accommodative lenses seem to be affected by capsular thickening, which is worse in pseudoexfoliation patients, causing aberrant folding of the lens known as “Z-Syndrome” [69].

## 7. Conclusions

Cataract surgery in patients with glaucoma generates many considerations for the surgeon who seeks to prevent the possible additional complications and to take advantage of the favorable results. Avoidance of postoperative IOP spikes would protect many glaucomatous eyes from loss of visual fields, and timely use of cataract surgery could reduce the need for IOP lowering medication. Selection of type and time of operation must offer the highest amount of benefit without compromising the potential glaucoma surgery in the future.

Cataract surgery in a patient with a previous trabeculectomy certainly is more complicated in relation to the technique used, as it is widely thought to cause bleb failure. In order to avoid bleb scarring, it is advisable to wait for about two years after the trabeculectomy, if at all possible. Prior to the operation the functionality of the bleb should be checked, either by simply measuring the pressure and assessing its morphology or by using AS-OCT and UBM [45, 46]. If it is found to be malfunctioning and a combined bleb revision/phacoemulsification procedure should be planned [44, 47]. At the time of surgery, extra care must be taken to perform an atraumatic procedure and avoid placing incisions over the bleb (both main and side ports) [47].

The management is relatively less complicated when the newer MIGS procedures are paired with cataract surgery. These devices have given a solution to those patients who have mild to moderate disease but are still uncontrolled using maximum drug treatment, are unable to tolerate it, or have other compliance issues.

It is understandable that glaucoma patients will want the best possible visual outcome following their cataract surgery and as such they will inquire or even research independently about toric and premium lenses. Toric lenses have indeed proven to improve refractive outcomes in glaucomatous patients [68], but it is more complex about premium lenses as they invariably either decrease contrast sensitivity or do not affect it at all. The only category that seems to benefit is glaucoma suspects or ocular hypertension patients without visual field defects and disc damage, or patients with very early damage which is unlikely to progress [69]. Overall cataract surgery in the glaucomatous patient is a challenging feat, but appropriate steps can be taken for the benefit of the patients to enjoy a fulfilling and beneficial outcome.

## Data Availability

The data used to support the findings of this study are available from the corresponding author upon request.
